# Cardiac magnetic resonance strain imaging in systemic light chain amyloidosis

**DOI:** 10.1186/1532-429X-16-S1-P321

**Published:** 2014-01-16

**Authors:** Florian Andre, Rebekka Kammerer, Kristin Breuninger, Arnt V Kristen, Christian Galuschky, Stefan Schoenland, Ute Hegenbart, Evangelos Giannitsis, Grigorios Korosoglou, Henning Steen, Hugo A Katus, Sebastian Buss

**Affiliations:** 1 Department of Cardiology, University of Heidelberg, Heidelberg, Germany; 2 TomTec Imaging Systems GmbH, Unterschleissheim, Germany; 3 Department of Hematology and Oncology, University of Heidelberg, Heidelberg, Germany

## Background

Systemic light chain amyloidosis (AL) is associated with a high cardiovascular morbidity and mortality. Cardiovascular involvement and determination of prognosis is underestimated by standard imaging parameters. Recently, cardiac deformation analysis of global circumferential and longitudinal strain has been shown to have great clinical impact on the assessment of prognosis and survival in this rare disease. For further analysis we applied the novel non-invasive post-processing feature tracking imaging (FTI) algorithm on pre-acquired regular CMR SSFP images in healthy volunteers and in patients with AL and sought to investigate different strain patterns.

## Methods

87 patients (mean age 60 ± 11 years) with biopsy proven systemic AL were scanned on a clinical 1.5 T CMR scanner (Philips Achieva). Short axis slices covering entirely both ventricles as well as 2-, 3- and 4-chamber views were acquired using standard SSFP-sequences before initiation of any specific pharmaceutical AL therapies. The control group consisted of 47 healthy subjects (mean age 59 ± 5 years). Besides the standard CMR parameters for volumes, ejection fraction, myocardial mass and wall thickness we measured the circumferential and the longitudinal strain on cine SSFP images by the application of the post-processing FTI algorithm.

## Results

In patients with AL mean longitudinal strain from the four chamber view (-15.9 ± 5.6% vs -21.3 ± 4%, p < 0.05) as well as midventricular mean circumferential strain (-22.8 ± 6.7% vs -25.1 ± 4.5%, p < 0.05) were significantly reduced compared to healthy subjects. Global circumferential strain and global longitudinal strain correlated with left ventricular ejection fraction (r = -0.61, p < 0.05; r = -0.62, p < 0.05). In the subgroup analysis of AL patients with a mean wall thickness less than 12 mm global longitudinal strain showed significantly reduced values in comparison to healthy control subjects (-18.3 ± 5.3% vs -21.3 ± 4%, p < 0.05), whereas global circumferential strain did not show a significant difference. Patients with an ejection fraction ≥55% already had reduced global longitudinal strains (-18 ± 4.7% vs -21.3 ± 4%, p < 0.05), again global circumferential strain did not show a significant difference.

## Conclusions

FTI strain analysis derived from regular cine SSFP sequences offers the possibility for a fast quantitative assessment of myocardial strain imaging patterns without additional and time-consuming strain imaging sequences. FTI strain analysis provides important insight into the disturbed contraction in AL. Further investigations are necessary to analyze the impact of this new method on the prediction of clinical outcome in AL patients.

## Funding

None.

